# Multi-Omics Integration Reveals Key Genes, Metabolites and Pathways Underlying Meat Quality and Intramuscular Fat Deposition Differences Between Tibetan Pigs and Duroc × Tibetan Crossbred Pigs

**DOI:** 10.3390/ani16020214

**Published:** 2026-01-11

**Authors:** Junda Wu, Qiuyan Huang, Baohong Li, Zixiao Qu, Xinming Li, Fei Li, Haiyun Xin, Jie Wu, Chuanhuo Hu, Sen Lin, Xiangxing Zhu, Dongsheng Tang, Chuang Meng, Zongliang Du, Erwei Zuo, Fanming Meng, Sutian Wang

**Affiliations:** 1 State Key Laboratory of Swine and Poultry Breeding Industry, Guangdong Provincial Key Laboratory of Animal Breeding and Nutrition, Institute of Animal Science, Guangdong Academy of Agricultural Sciences, Guangzhou 510640, China; 2 Guangxi Key Laboratory of Animal Breeding, Disease Control and Prevention, College of Animal Science and Technology, Guangxi University, Nanning 530004, China; 3 Sericultural & Agri-Food Research Institute, Guangdong Academy of Agricultural Sciences, Guangzhou 510640, China; 4 School of Medicine, Foshan University, Foshan 528051, China; 5 Jiangsu Key Laboratory of Zoonosis, Yangzhou University, Yangzhou 225009, China; 6 State Key Laboratory of Genome and Multi-omics Technologies, Shenzhen Branch, Guangdong Laboratory of Lingnan Modern Agriculture, Key Laboratory of Gene Editing Technologies (Hainan), Ministry of Agriculture and Rural Affairs, Agricultural Genomics Institute at Shenzhen, Chinese Academy of Agricultural Sciences, Shenzhen 518000, China

**Keywords:** pork quality, IMF deposition, local pig breeds, multi-omics

## Abstract

The sensory quality of pork constitutes a complex phenotype that arises from the interplay between genetic factors and environmental conditions. As a local pig breed in China, Tibetan pigs (TPs) are known for their high-quality meat. However, their slow growth rate and low production efficiency limit their large-scale breeding. We have used Duroc as a hybrid sire to improve TP. Our study found that TPs have higher intramuscular fat content and higher levels of monounsaturated fatty acids. Duroc × Tibetan crossbred pigs (DZs) not only retain the paternal high productivity but also inherit the superior meat quality of the maternal parent. Transcriptome analysis identified *IL6*, *GPX1*, *GPX3*, *AOX1*, *ALDH7A1*, *PTGS2*, *NFKBIA*, *ADIPOQ* and *PPARG* as being involved in affecting meat quality. Metabolomic analysis found that betaine, carnosine, L-carnitine, and lysophosphatidylcholine were important components that affect meat quality. Joint analysis further reveals that the expression of *ATF4*, *DGKB*, *GNMT*, and *ADSL* genes is closely related to arachidonic acid, lysophosphatidylcholine, betaines, and hypoxanthine, ultimately affecting the quality of the meat. By comprehensively analyzing the carcass and meat quality traits, genes and metabolites affecting meat quality traits, this study provides new evidence for improving pork quality and guiding breeding strategies.

## Simple Summary

Tibetan pigs are a valuable local pig breed in China, known for their excellent meat quality. However, their slow growth and low production efficiency limit large-scale breeding. Crossbreeding with Duroc pigs is a promising strategy to balance meat quality and production performance. Yet, the molecular mechanisms underlying the differences in meat quality and intramuscular fat deposition between TPs and DZs remain unclear. Our study addresses this gap through phenotype detection and multi-omics analysis.

## 1. Introduction

Pork is one of the most widely consumed meats in the global market. As the economic standards of residents continue to rise, consumers are beginning to pay more attention to the meat quality. The colour, textural characteristics (e.g., intramuscular fat marbling) and tenderness characteristics of meat all influence consumers’ purchasing decisions. Actually, these quality-related factors also determine the texture perception and nutritional worth of pork [1]. Mechanistically, meat quality is determined by genetics, breeds, feed, rearing methods, environment, slaughtering, and processing [2]. Among these, the contribution of genes to the meat quality ranges from 10% to 70% [3]. Even with an ideal diet, favourable rearing conditions, and advanced meat processing techniques, insufficient genetic potential will still compromise pork quality [4,5]. Many local pig breeds exhibit superior meat quality traits due to their unique genetic background. However, many local breeds often have undesirable production traits [6]. As a local breed in China, TPs are famous for their excellent meat quality characteristics. Studies have reported that the intramuscular fat content of TPs is higher than that of commercial lean-type breeds, including Landrace and Yorkshire pigs [7]. Additionally, compared to lean-type breeds, the content of monounsaturated fatty acids (MUFAs) in the longissimus dorsi muscle of TPs is also higher [8]. These superior meat traits have contributed to their high market acceptability.

As an effective genetic improvement strategy, crossbreeding has been widely applied for such a long time [9]. We crossbred local pig breeds (Tibetan pig) with lean-meat breeds (Duroc pig) to obtain the advantages of both parents simultaneously. Zhang et al. [10] found that the F1 generation derived from the cross between Jiaxing Black pigs and Yorkshire pigs exhibits distinct advantages, including improved high-quality traits, enhanced slaughter performance, and more favourable flavour compounds. These hybrid offspring not only retain the paternal (Yorkshire pigs) advantages (rapid growth and high productivity) but also the excellent meat quality of the maternal parent (Jiaxing Black pigs). Li et al. [11] evaluated the growth performance and meat quality of Berkshire × Chenghua crossbred pigs. They found that the crossbred pigs showed significantly improved growth rates and meat quality, with significant increases in intramuscular fat (IMF) content and amino acid composition. The Duroc pig is regarded as an ideal parent breed for enhancing the production performance of local pigs due to its rapid growth rate, high feed conversion efficiency, and superior carcass quality [12]. Rodrigues Dos Santos et al. found that the hybrid individuals with a higher genetic contribution from the Duroc breed exhibited superior growth performance and improved carcass quality [13]. Another research conducted by Terada et al. revealed that the Duroc × Jinhua crossbred pigs exhibited significant heterosis in growth rate and lean meat percentage [14]. Given that the genetic material of Duroc pigs has a positive impact on these breeding systems, we infer that Duroc × Tibetan crossbred pigs exhibit superior production performance while maintaining high-quality meat characteristics.

With the rapid development of multi-omics technologies, researchers have utilized them to explore the unique regulatory mechanisms underlying meat traits. For example, by combining transcriptomics with targeted metabolomics, Zhen Luo et al. [15] explore the mechanism of the development of the longissimus dorsi muscle in different pig breeds. This study revealed that changes in genes and metabolites related to fatty acid biosynthesis and choline metabolism had a significant impact on meat quality characteristics. Moreover, Kumar S.T. et al. found that fatty acid β-oxidation and amino acid metabolism pathways were crucial for enhancing meat tenderness and flavour by analyzing the transcriptome and metabolome of local fat-type pigs and lean-type pigs [16]. Our study explored the differences between TPs and Duroc–Tibetan (DZ) crossbreds by examining carcass and meat quality characteristics. Meanwhile, we also used transcriptomics and metabolomics to investigate gene expression patterns, metabolic pathways, and regulatory networks in the muscle of TPs and DZs. We aim to identify key genes and metabolites that may be potential targets for improving pork quality. Additionally, developing the local pig breeds industry can also facilitate the diversification of market products and support the sustainable development of the animal husbandry industry.

## 2. Materials and Methods

### 2.1. Animals and Sample Preparation

To compare the growth performance, carcass performance, and meat quality of the TP group and DZ group, we selected 6 eight-month-old female Tibetan pigs and 6 eight-month-old female Duroc–Tibetan hybrids (240 days from birth to slaughter). Animals were reared under the same conditions with natural, uncontrolled room temperature and light. All experimental pigs were housed in groups and fed with free access. The final body weights of TPs were (38.93 ± 1.99) kg, and the final body weights of DZs were (66.4 ± 13.02) kg. Animals were slaughtered according to the national slaughter standards. Subsequently, the carcass performance and meat quality of the pigs were tested. Fresh longissimus dorsi muscle samples were preserved in 4% paraformaldehyde solution. All samples were stored at −80 °C for examination of fatty acids, transcriptomic, and metabolomic profiles.

### 2.2. Carcass and Meat Quality Performance

After slaughter, the carcass and meat quality performances of the TP group and DZ group were determined according to national measurement standards (Criterion Code: NY/T 1333-2007) [17].

### 2.3. Preparation of Adipose Tissue Sections and Cell Size Measurement

Samples were sent to Changsha Weiser Biological Co., Ltd. (Weiser Biological Cooperation, Changsha, China) for the preparation of tissue slices. Photographs were taken under 100× (10× objective lens and 10× eyepiece lens) and 400× (40× objective lens and 10× eyepiece lens) magnifications.

### 2.4. Determination of Fatty Acids

At least 150 mg of intramuscular fat was extracted from each sample for gas chromatograph analysis. First, the sample was dissolved in a mixed solution consisting of triethanolamine, gallic acid, zeolite, 95% ethanol and water. Subsequently, the purified substance from the previous step was saponified with sodium hydroxide in methanol and boron trifluoride in methanol using a condenser and water bath. The purified fat was then reacted with heptane solution, saturated sodium chloride solution, and anhydrous sodium sulfate. Finally, the sample was tested using a gas chromatograph (Agilent Technologies, Santa Clara, CA, USA). The conditions were as follows: capillary column length, 100 m, inner diameter, 0.5 mm and membrane thickness, 0.25 μm. Detection temperature: 280 °C. Temperature program: 100 °C (13 min) → 10 °C/min to 180 °C → 1 °C/min to 200 °C → 4 °C/min to 240 °C (10.5 min). Nitrogen was used as the carrier gas with a split ratio of 50:1.

### 2.5. Transcriptome Sequencing and Bioinformatics Analysis

Total RNA was extracted from the tissue samples using TRIzol reagent (Tiangen, Beijing, China) according to standard protocols. The quality and concentration of the extracted RNA were determined using an Agilent 5300 Bioanalyzer (Santa Clara, CA, USA) and a NanoDrop ND-2000 spectrophotometer, respectively. Only RNA samples meeting strict quality standards were selected for subsequent library construction. The sequencing library was developed by Shanghai Meiji Biotechnology Co., Ltd. (Shanghai, China) and constructed and sequenced using Illumina’s Stranded mRNA Prep, Ligation Kit (San Diego, CA, USA). After quantification with a Qubit 4.0 fluorometer (Thermo Fisher Scientific, Waltham, MA, USA), the library was sequenced on the NovaSeq X Plus platform (PE150) using NovaSeq Reagent Kits (Illumina, San Diego, CA, USA). Raw paired-end reads were trimmed and subjected to quality control using fastp software (v0.26.0, https://github.com/OpenGene/fastp, accessed on 27 November 2025) with default parameters. HISAT 2 (v2.2.1, http://ccb.jhu.edu/software/hisat2/index.shtml, accessed on 27 November 2025) is then used to compare the clean reads to the reference genome. StringTie (v2.2.1, http://ccb.jhu.edu/software/stringtie/, accessed on 27 November 2025) is used for transcript assembly, using a method guided by the reference genome. For differential expression analysis, we used the TPM (Transcripts Per Million) method to estimate transcript abundance and RSEM (v1.3.3, https://deweylab.github.io/RSEM/, accessed on 27 November 2025) to quantify geneexpression. DESeq2 (v1.42.0, https://bioconductor.org/packages/release/bioc/html/DESeq2.html, accessed on 27 November 2025) is primarily used to identify differentially expressed genes (DEGs), with a significance criterion of |log2FC| ≥ 1 and FDR < 0.05. Functional enrichment analysis of DEGs was performed, which identified a large number of GO terms and KEGG pathways. Statistically significant enrichment results were obtained relative to the entire transcriptome (Bonferroni-corrected *p* < 0.05) using Goatools (v0.12.5, https://github.com/tanghaibao/Goatools, accessed on 27 November 2025) and Python’s SciPy package (v1.12.0, https://scipy.org/, accessed on 27 November 2025).

### 2.6. Untargeted Metabolite Assay and Bioinformatics Analysis

First, samples were homogenized using a Wonbio-96C cryogenic tissue grinder (Shanghai Wanbo Biotechnology Co., Ltd., Shanghai, China), followed by low-temperature ultrasonic extraction. After the mixture was allowed to stand and centrifuged, the supernatant was carefully transferred to a sample vial for subsequent LC-MS/MS analysis. To ensure data stability, all samples are mixed in the same volume to create quality control (QC) samples. These QC samples were processed and analyzed alongside the experimental samples to verify the stability of the method throughout the experimental process. LC-MS/MS analysis was performed at MajorBio Biotechnology Co., Ltd. (Shanghai, China) using a UHPLC-Orbitrap Explois 240 system. Metabolites identification mainly relies on database comparisons, using HMDB (https://hmdb.ca/), Metlin (https://metlin.scripps.edu/, accessed on 27 November 2025), and the MJDB database. The data matrix is uploaded to MajorBio Cloud Platform (https://cloud.majorbio.com) for processing. The standard data set was generated by adjusting the spectral peak intensities using the summation normalization method. The QC samples were then filtered to remove variables with relative standard deviations (RSD) greater than 30%, resulting in a cleaner data matrix for subsequent analysis. PCA and OPLS-DA analyses were performed using the “ropls” R package (v1.1.6.2.2), and model reliability was checked by three-fold cross-validation. To be statistically significant, metabolites must meet two conditions at the same time: VIP value of OPLS-DA > 1, and *p*-value of Student’s *t*-test < 0.05.

### 2.7. Combined Transcriptome and Metabolome Analysis

Python’s scipy library (version 1.0.0), OmicsPLS (v2.0.2) and the vegan package (v2.6.4) were used to analyze the relationship between DEGs and DMs. Subsequently, the identified differentially expressed genes (DEGs) and differentially expressed metabolites (DEMs) were mapped to the KEGG database, followed by pathway enrichment analysis to identify biologically meaningful interactions. Pathways with statistical significance (*p* < 0.05) were designated as key gene–metabolite association pathways. All computational work was performed on the Major Cloud platform (v4.5.0).

### 2.8. qRT-PCR Analysis

Total RNA was extracted from animal tissues using the Tiangen RNA Extraction Kit (Tiangen, Beijing, China). The concentration and purity of the RNA were measured using a NanoDrop 2000 spectrophotometer (Thermo Fisher Scientific, Waltham, MA, USA). Subsequently, 1% agarose gel electrophoresis was performed to verify the completeness of the RNA. The prepared RNA samples were immediately frozen and stored at −80 °C for later use. The cDNA was synthesized using HiScript III All-in-one RT SuperMix for qPCR (Vazyme, Nanjing, China). The prepared cDNA is stored at −20 °C. The PCR reaction included an initial denaturation step at 95 °C for 30 s, followed by 40 cycles of 95 °C for 10 s and 60 °C for 30 s. The 2^−ΔΔCt^ method was used to calculate the expression of each gene. Primer sequences are shown in Table S1.

### 2.9. Statistical Analysis

All statistical organization and analysis of experimental data were conducted using Excel 2013 software. The independent samples *t*-test was employed to analyze the differences in mean values between the two groups. Analysis results are presented in the text in the form of means and standard deviations, and differences are considered significant when *p* < 0.05.

## 3. Results

### 3.1. Carcass and Meat Quality Characteristics of Pigs

Firstly, we found significant differences in the slaughter and meat quality traits between TPs and DZs (Table 1). Specifically, the DZ group exhibited a higher live weight (66.4 ± 13.02 kg vs. 38.93 ± 1.99 kg) and carcass weight (43.9 ± 8.17 kg vs. 25.51 ± 4.68 kg) (*p* < 0.05). Furthermore, the DZs exhibited greater skin thickness (4.05 ± 0.26 mm vs. 3.92 ± 0.31 mm) and a larger loin eye area (40.65 ± 2.76 cm^2^ vs. 17.11 ± 3.08 cm^2^), indicating a higher production capacity. In terms of meat quality, post-slaughter pH values after 24 h in TPs were slightly higher (5.86 ± 0.07 vs. 5.51 ± 0.01) (*p* < 0.05). Colour analysis showed that the meat of DZs was lighter (45.59 ± 8.65 vs. 49.42 ± 14.16) and less red (11.53 ± 5.42 vs. 14.54 ± 5.26) (*p* < 0.05). Moreover, the cooking loss rate in the DZ group was significantly reduced (32.41 ± 2.01% vs. 36.83 ± 2.79%). In addition, the IMF content of TPs was 3.73 ± 0.13%, which was significantly higher than that of the DZ group (2.20 ± 0.41%, *p* < 0.05).

### 3.2. Intramuscular Fatty Acid Composition and Content in Pigs

**Figure 1:**
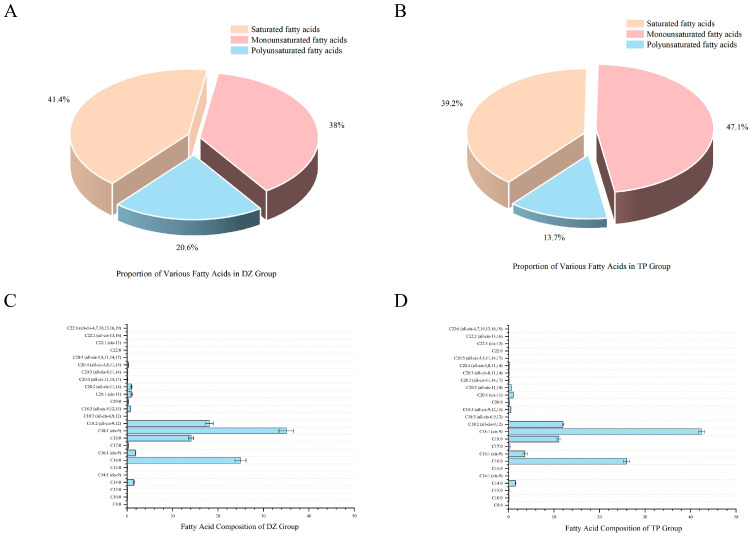
Content of various fatty acids: (**A**) Composition within the interfacial film framework (DZ), (**B**) Composition within the interfacial film framework (TP), (**C**) Content of Fatty Acids in DZ, (**D**) Content of Fatty Acids in TP.

To gain a deeper understanding of meat quality, we continued to measure the specific IMF composition in TPs and DZs. The IMF composition of the TP group mainly consisted of monounsaturated fatty acids (MUFA) (47.1%), followed by saturated fatty acids (SFA) (39.2%) and polyunsaturated fatty acids (PUFA) (13.7). However, in the DZ group, the main components of IMF were SFA (41.4%), followed by MUFA (28%) and PUFA (20.6%) (Figure 1A,B). The contents of 25 different fatty acids were further detected (including 10 SFA, 10 PUFA, and 5 MUFA). Palmitic acid (C16:0) was the most abundant fatty acid among the SFA. The palmitic acid content in the TP group reached 25.95 ± 0.726%, higher than that in the DZ group (24.95 ± 1.22%) (*p* < 0.05). Among MUFA, oleic acid (C18:1(cis-9)) exhibited the highest content in both groups. However, the TP group showed a significantly higher level (42.39 ± 0.696%) compared to the DZ group (35.014 ± 1.587%) (*p* < 0.05). Among PUFA, linoleic acid (C18:2(all-trans-9, 12)) was the predominant component. Linoleic acid content in the DZ group (18.099 ± 0.836%) is significantly higher than that in the TP group (11.974 ± 0.176%) (*p* < 0.05).

### 3.3. Comparison of Intramuscular Adipocyte Morphology

**Figure 2:**
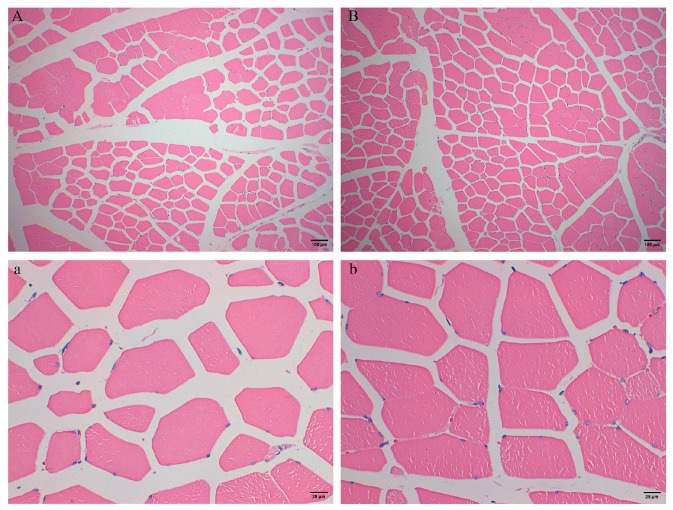
H&E-stained images of intramuscular adipose tissue in the TP and DZ muscles. (**A**,**a**) 100× and 400× magnified images of IMF tissue sections from the TP group. (**B**,**b**) 100× and 400× magnified images of IMF tissue sections from the DZ group.

The location and pattern of fat deposition are also important factors affecting meat quality. Our data revealed differences in the shape and size of adipocytes in muscle between the TP and DZ. As shown in Figure 2, viewed at 100 (10× objective lens and 10× eyepiece lens) and 400 (40× objective lens and 10× eyepiece lens) times, TPs have larger fat droplets and looser cell structure (Figure 2A,a), while DZs have smaller adipocytes and more tightly arranged fat droplets (Figure 2B,b). These findings suggest that TPs exhibit richer IMF deposition, which may be related to their unique pork quality.

### 3.4. Transcriptomic Analysis

**Figure 3:**
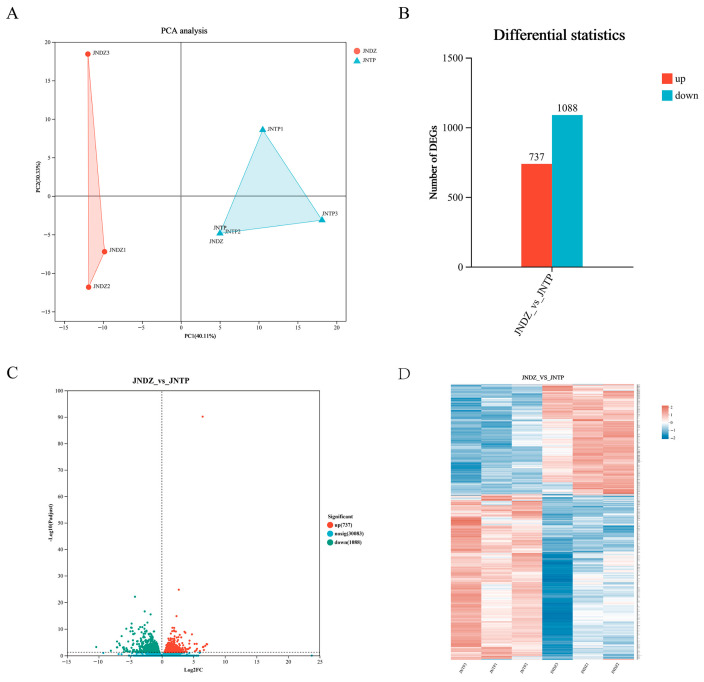
Differential Transcriptomic Analysis. (**A**) PCA Analysis of TP and DZ Groups. (**B**,**C**) Volcano map and column graph of upregulated and downregulated genes in TP and DZ groups. (**D**) Systematic cluster analysis of the DEGs in TPs and DZs.

The study performed sequencing on a high-throughput sequencing platform, and each sample set produced at least 45.77 million raw reads. However, these initial data contain interfering substances such as adapter sequences, low-quality fragments, fuzzy bases (high N content), and excessively short fragments- all of which will affect subsequent analysis. To ensure the reliability of data, we strictly filtered the raw data and obtained high-quality sequences suitable for further processing. As shown in Table S2, more than 97.46% of the raw readings were retained as Clean Reads, with an average GC content of 51.14% and a Q30 score of over 93.89%. PCA revealed a distinct separation and clustering of samples derived from the TP and DZ groups (Figure 3A). In a preliminary study of the differential gene expression patterns in IMF between TPs and DZs, the differentially expressed genes were identified with |log2FC| ≥ 1 & *p* < 0.05, a total of 1825 differentially expressed genes (DEGs) were identified, comprising 737 upregulated genes (40.4%) and 1088 downregulated genes (59.6%) (Figure 3B,C). The clustered heatmap of DEGs illustrates distinct patterns in gene expression levels (Figure 3D). This analysis helps us to understand the relationships and interactions among these DEGs in depth.

### 3.5. GO and KEGG Analysis of Differentially Expressed Genes

**Figure 4:**
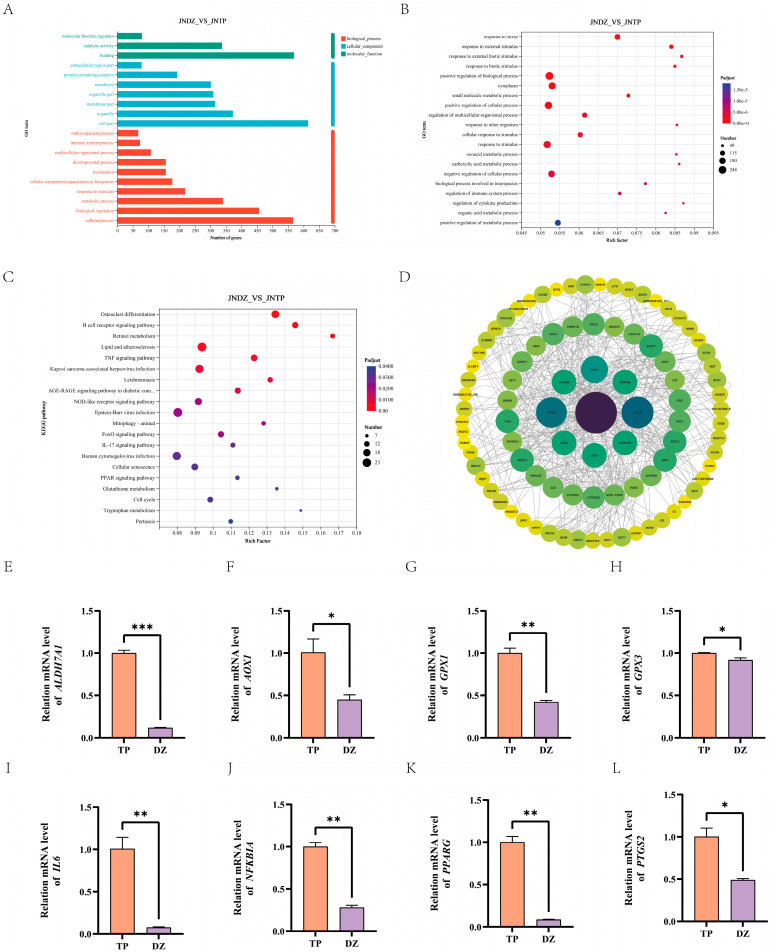
Enrichment analysis and verification of differentially expressed genes. (**A**) Analysis of GO Annotations for DEGs Between TPs and DZs. (**B**) The scatter plot shows the top 20 GO entries. The larger the enrichment factor, the more important it is, and the larger the dots, the more enriched the DEGs in the pathway are. (**C**) Scatter plot showing the enrichment analysis of the top 20 KEGG pathways. A larger Rich factor indicates higher enrichment. Larger points indicate a higher number of DEGs enriched in that pathway. (**D**) Visualization of protein–protein interaction network analysis. (**E**–**H**) Real-time quantitative PCR results showed the expression profiles of *ALDH7A1*, *AOX1*, *GPX1*, and *GPX3* in TPs and DZs. (**I**–**L**) The expression of *IL6*, *NFKBIA*, *PPARG*, and *PTGS2* in TP and DZ groups. Statistical significance indicated: (* *p* < 0.05, ** *p* < 0.01, *** *p* < 0.001).

To further clarify the functional roles of differentially expressed genes across various groups, we conducted a comprehensive Gene Ontology (GO) functional annotation analysis. GO analysis categorizes gene functions into three major categories: biological processes, cellular components, and molecular functions. Among them, the gene enrichment was most prominent in the biological process. The top five categories were cellular processes, biological regulation, metabolic activities, response to stimuli, and cell composition/biogenesis. Next are cellular components, primarily including cell structure, organelles, membrane components, and organelle components. Molecular functions were significantly enriched in three aspects: molecular binding, enzyme activity, and molecular function regulation (Figure 4A). Further GO enrichment analysis found that biological processes related to lipid metabolism are critical, especially small molecule metabolism (GO:0044281), organic acid metabolism (GO:0006082), and carboxylic acid metabolism (GO:0006084) (Figure 4B). These biological processes are essential in the synthesis, decomposition, and transformation of lipids. Analysis of the KEGG pathway of DEGs found that they were involved in 318 different metabolic pathways. The KEGG enrichment plot shows the ten most important pathways (Figure 4C). We focused on genes related to lipid metabolism, such as *IL6*, *GPX1*, *AOX1*, *ALDH7A1*, *PTGS2*, *ADIPOQ*, and *PPARG*, and built a protein interaction network (PPI) (Figure 4D). RT-qPCR later verified the expression pattern of these genes (Figure 4E–L), which verified the reliability of transcriptome analysis. These results suggest that genes involved in fatty acid and lipid metabolism pathways are likely candidate genes for IMF deposition, which may contribute to the higher IMF content in TPs. Importantly, differential gene expression in genes associated with lipid and energy metabolism may lead to differences in fat deposition capacity between TPs and DZs.

### 3.6. Metabolomics Results and Analysis

**Figure 5:**
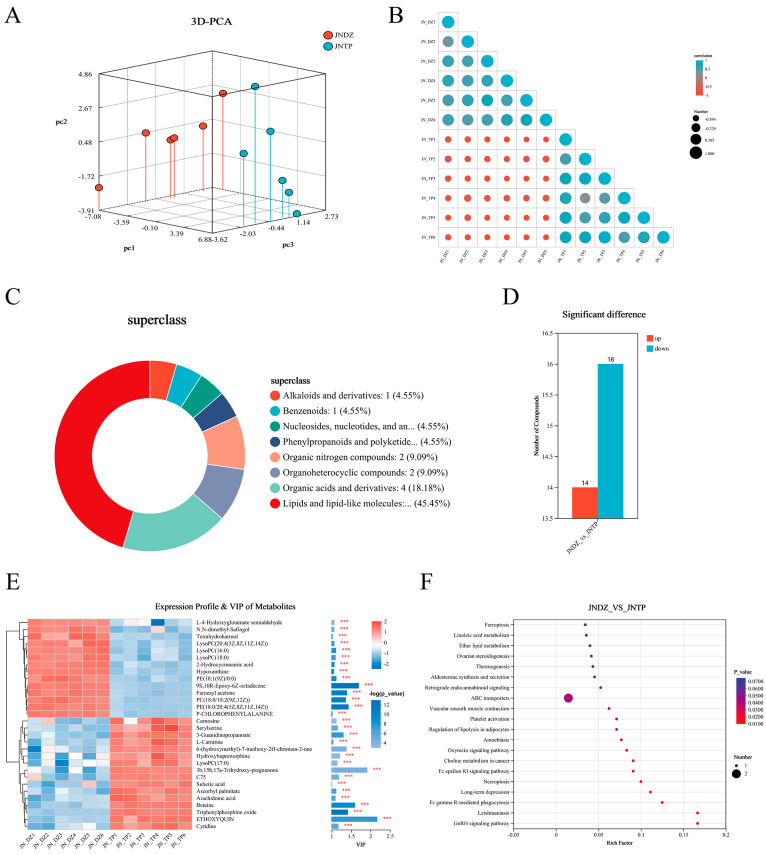
Metabolomic analysis. (**A**) PCA Analysis Plot of Samples. (**B**) Heat Map of Correlation Analysis. (**C**) HMDB Classification Ring Diagram. (**D**) Bar Chart of Differential Metabolite Analysis. (**E**) Differential Metabolite Cluster Analysis and VIP Value Analysis Plot (*** *p* < 0.001). (**F**) KEGG Enrichment Analysis Plot of DMs.

Heatmaps and principal component analysis revealed significant differences between the samples of different groups. The results indicate a significant distinction between the TP and DZ groups, while the differences within each group are not significant (Figure 5A,B). These differential metabolites were predominantly lipid-related compounds (45.45%), followed by organic acids (18.18%), heterocyclic compounds (9.09%), nucleoside derivatives (4.55%), phenylpropanoids (4.55%), aromatic compounds (4.55%), and alkaloids (4.55%) (Figure 5C). Applying stringent criteria (fold change ≥ 1, VIP score ≥ 1, *p* < 0.05), 30 metabolites showed significant differences between the DZ and TP groups—14 increased and 16 decreased (Figure 5D). VIP scores indicated betaine, phosphatidylcholine, arachidonic acid, carnosine, and hypoxanthine as the top five lipid-related metabolites (Figure 5E). KEGG pathway analysis mapped these metabolites to 34 biological pathways, of which 20 were significantly enriched (*p* < 0.05). Furthermore, eight pathways are directly involved in lipid metabolism, including linoleic acid metabolism, ether lipid synthesis, steroid production, body temperature regulation, aldosterone signalling, lipolytic regulation, and oncogenic choline metabolism (Figure 5F).

### 3.7. Joint Analysis of Differential Metabolites (DMs) and DEGs

**Figure 6:**
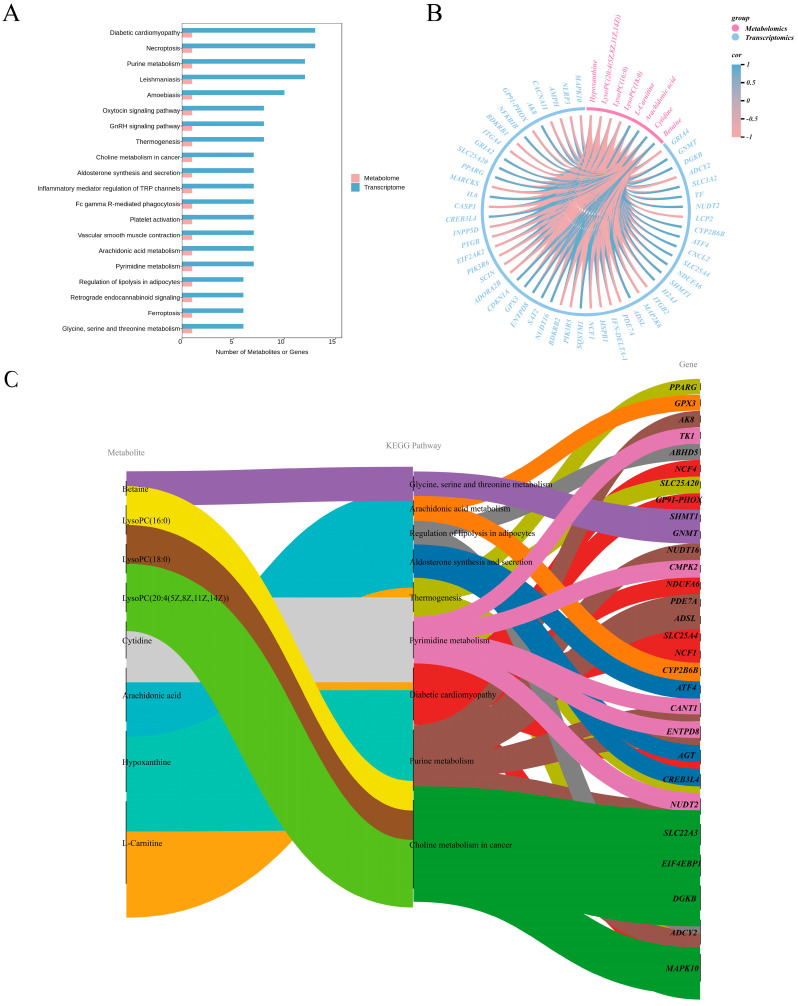
Integrated analysis of metabolomics and transcriptomics data. (**A**) Common Prosperity Collection Channel Bar Chart. (**B**,**C**) String Diagram and Mulberry Diagram of Correlations Between Genes and Metabolites.

To study the relationship between DEGs and DMs and meat quality, as well as the metabolic pathways shown in Figure 6, we performed a KEGG pathway analysis of DEGs and DMs in the DZ and TP. We identified 20 co-enriched core pathways and visualized them (Figure 6A). Nine of these pathways are involved in lipid metabolism, and the related genes and metabolites are listed in Table S3. It is particularly noteworthy that arachidonic acid derivatives are involved in multiple pathways, while other metabolites, such as L-carnitine, beta, and lysophosphatidylcholine, also play roles in distinct metabolic processes. Subsequently, Pearson correlation analysis was conducted on DEGs and DMs in these co-enriched pathways. Correlation string plots and mulberry plots were used to visualize the relationships between metabolites and genes (Figure 6B,C). Thirty genes are positively correlated with metabolites such as arachidonic acid, beta, cytosine, and L-carnitine, while 23 genes are negatively correlated. In addition, sphingomyelin (18:0, 16:0, and 20:4 (5Z, 8Z, 11Z, 14Z)) and hypoxanthine were negatively associated with 30 genes and positively associated with 23 genes. *SLC3A2*, *NUDT2*, *DGKB*, *HA2J*, *NDUFA6*, *ADSL*, *SQSTM1*, *ATF4*, *SLC25A4*, and *GNMT* genes are strongly positively correlated with the metabolites of arachidonic acid, beta, cytosine, and L-carnitine. In contrast, *LCP2*, *ADCY2*, *GRIA4*, *ADORA2B*, *NCF1*, *ITGB2*, and *PIK3R5* genes were significantly negatively correlated with lysosphatidylcholine (18:0, 16:0, and 20:4 (5Z, 8Z, 11Z, 14Z)) and hypoxanthine. These findings suggest that these DMs may be determined by DEGs, which in turn lead to differences in the meat quality phenotype.

## 4. Discussion

Driven by the emphasis on pork quality, consumers are paying increasing attention to local pig breeds. Although TPs have the advantage of high-quality meat, their slow growth rate and low production efficiency limit their large-scale breeding. To increase the utilization rate of TPs, we selected Duroc pigs as the crossbreeding boars to enhance their production performance. Meanwhile, we compared the differences between TPs and DZs in carcass and meat quality traits. Furthermore, we also explore the mechanisms underlying the differences in IMF deposition among them by using multi-omics analysis. These findings provide important evidence for improving pork quality.

Our results showed that the live weight, body length, loin eye area, and backfat thickness of Duroc–Tibetan crossbred pigs are significantly better than those of TPs. These findings can be attributed to the growth-associated candidate genes present in Duroc pigs, which improve the growth performance of DZs through dominant effects [18]. A previous study has also reported that the Duroc–Meishan crossbred pigs had superior growth performance and improved carcass quality [19]. The pH value of the meat is another important factor in evaluating meat quality. The glycogen stored in the muscle will be converted into lactic acid after the animal is slaughtered. Subsequently, the accumulation of lactic acid decreases the pH value of the meat. After slaughter, the pH of pork decreases from an initial value of 6.8 to a range of 5.4–5.7. The enhanced glycogen metabolism will increase lactic acid content, which in turn decreases the meat pH value [20]. In our study, the meat pH values of TPs and DZs at 24 h post-slaughter were 5.51 ± 0.01 and 5.86 ± 0.07, respectively. Notably, the meat pH value of TPs after 24 h was lower. TPs’ unique physiological characteristics may cause this result. The unique hypoxic adaptation ability of TPs stems from its faster energy metabolism capacity. This ability results in a faster decline in the pH value of the meat after slaughter [21]. The 24 h pH value of DZs is higher than the normal range, which may be attributed to the incomplete post-mortem glycolysis in their muscle tissue.

Meat colour is another key factor that affects pork quality. Our results revealed that both TPs and DZs had excellent lightness (L value) and yellowness (B value). Notably, the meat colour (redness) of TPs was higher (14.65 ± 2.04) than that of DZs (11.69 ± 2.03). This result is associated with high myoglobin content in TPs. Higher myoglobin content indicates a stronger oxygen-binding capacity. As a local breed living in a high-altitude area, TPs have elevated myoglobin levels, resulting in a redder meat colour [21]. The IMF content in pork has a significant impact on its quality. The amount of IMF reflects the tenderness, juiciness, and flavour of meat [22]. The IMF content in the TP longissimus dorsi muscle is about 3.73 ± 0.13%, which is significantly higher than that of DZs (2.20 ± 0.41%). Although the high intramuscular fat content of TPs may enhance meat flavour, their significantly higher cooking loss rate suggests that, compared to Duroc×Tibetan pig hybrids, TP meat may exhibit reduced juiciness after cooking. Furthermore, the IMF content of DZs was still higher than that of the commercial foreign three-way crossbred pigs. Previous studies have reported that the IMF content of 6-month-old and 10-month-old foreign three-way crossbred pigs is 1.02 ± 0.11% and 1.92 ± 0.08%, respectively [23]. These results indicate that TPs and DZs indeed exhibit advantages in intramuscular fat deposition. In conclusion, DZs not only retain the paternal (Duroc pigs) advantages (rapid growth and high productivity) but also the excellent meat quality of the maternal parent (TP).

The composition of FAs in pork is another key factor affecting meat nutrition and flavour. Generally, the FAs can be mainly classified into three categories: SFA, MUFA, and PUFA. Each category has a different impact on nutrition and health. SFA mainly provides energy and is involved in the formation of cell membranes. However, excessive intake of SFA will trigger cardiovascular disease [24]. MUFA can regulate blood lipids, reduce inflammation, maintain cell membrane integrity, and benefit metabolic health [25]. Adding PUFA helps inhibit inflammation, protect against cardiovascular diseases, promote brain health, and enhance immunity [26]. In this study, the SFA content in the IMF of TPs and DZs (40.30 ± 0.51%; 28.94 ± 0.65%) was lower than that of UFA (60.38 ± 1.94%, 69.81 ± 1.71%), indicating that TPs had superior meat flavour. The melting point of oleic acid is very low, which makes the fat easier to melt during cooking. Meanwhile, a positive correlation exists between the oleic acid content in muscle tissue and the juiciness of cooked pork, with elevated levels of this monounsaturated fatty acid contributing to enhanced juiciness of the final cooked product [27]. Furthermore, oleic acid produces special flavour substances when heated [28]. In this study, C18:1 (cis-9) oleic acid was identified as the most abundant unsaturated fatty acid in the intramuscular fat of both TPs (41.1 ± 1.156 g/100 g) and Duroc–Tibetan crossbred pigs (DZs, 23.476 ± 2.015 g/100 g). This finding provides a theoretical basis for the delicious taste of TPs’ and DZs’ meat.

In terms of tissue morphology, TPs exhibited larger fat droplets with a loose cellular architecture. All genetic factors, growth characteristics, and lipolytic enzyme activity cause this difference. Local pig breeds and commercial pigs also differ in mRNA levels of genes related to IMF deposition. Local pig breeds grow more slowly and have a lower lean meat percentage, while the mainstream commercial lean-meat pigs grow faster and have a higher lean-meat percentage. On the one hand, the slow growth pattern of local pig breeds may allow their adipocytes more time to develop, resulting in larger cell sizes [5]. On the other hand, local pig breeds have higher lipogenic enzyme activities (such as malate dehydrogenase, acetyl-CoA carboxylase, glucose-6-phosphate dehydrogenase, and fatty acid synthase) [29] and lower lipolytic enzyme activities (such as hormone-sensitive lipase) [30]. Consequently, local pig breeds exhibit enhanced lipogenesis and reduced lipolysis. These metabolic changes collectively lead to an increase in the size of intramuscular adipocytes in the local pig.

The IMF has long been recognized as a key factor in evaluating meat quality. Firstly, transcriptome analysis was performed, and differentially expressed genes were identified. Subsequent GO and KEGG enrichment analyses identified pathways closely associated with intramuscular fat deposition. By PPI network analysis, we found nine DEGs that may be involved in regulating fat deposition. The *IL-6* can not only inhibit genes related to fat synthesis in adipocytes (such as *PPARG* and *PLIN1*), reducing fat synthesis and storage, but also indirectly affect fat synthesis and storage by regulating insulin sensitivity [31]. Additionally, *IL-6* can regulate fatty acid oxidation in adipocytes [32]. Porcine *IL-6* gene expression levels were significantly correlated with IMF content [33]. In this study, the mRNA level of *IL-6* in TPs was higher than that detected in DZs. The *GPX1* and *GPX3* genes, respectively, encode glutathione peroxidase 1 and 3, which are key enzymes involved in the cellular antioxidant defence system. Previous studies have demonstrated that *GPX1* participates in lipid metabolism. Specifically, in studies focusing on chickens, the deposition of IMF is closely associated with pyruvate and citric acid metabolism, and the *GPX1* gene has been identified as one of the major genes regulating this process [34]. Currently, there are few studies on *GPX3*, and further exploration is needed. The *PTGS2* is another important gene that affects lipid metabolism. Overexpression of *PPARG* in TPs suppresses *PTGS2* expression, reduces PGE_2_ synthesis, and thereby promotes the diversion of arachidonic acid toward lipid synthesis pathways, leading to increased intramuscular fat deposition. *PTGS2* is a key downstream target of *PPARG* regulation in intramuscular fat [35]. In our research, the TPs had higher PTGS2 levels, which were consistent with their phenotypic traits. *PPARG* is the “commander-in-chief” of adipocyte differentiation and fat metabolism, which controls the growth of adipocytes and regulates fat deposition [36]. The bovine *PPARG* gene regulates fat accumulation through upregulating the circular RNAs (circPPARGs). Specifically, these circPPARGs interact with the *PPARG* protein, further inhibiting the functional activity of the *HSL* gene and enabling adipocytes to accumulate large amounts of lipids [37]. Our study indicated that the *PPARG* level in TPs is higher than that of DZs.

This study identified lipid-related compounds accounting for up to 45% of the differential metabolites, a finding closely linked to the fat deposition characteristics of TPs and DZs. As an excellent local breed in China, TPs exhibit high intramuscular fat content and abundant subcutaneous fat deposition. In contrast, Duroc crossbred pigs, bred primarily for growth performance, demonstrate relatively lower levels of fat accumulation. The innate differences in fat metabolism between breeds directly drive the differential accumulation of lipid-related metabolites, making them the most critical metabolic markers distinguishing the two sample groups. This finding also aligns with the previous transcriptomic analysis, which revealed significant enrichment of genes in lipid metabolism pathways, confirming the key role of lipid metabolism in regulating the differences in pork quality between the two breeds.

Metabolomic studies also identify various DMs. VIP analysis showed that four metabolites were highly correlated with IMF deposition, including beta, carnosine, L-carnitine, and lysophosphatidylcholine. Betaine is a naturally occurring compound found in all kinds of living organisms, which plays a crucial role in regulating fat deposition. Betaine participates in modulating gene expression and lipid metabolism pathways. Previous studies have demonstrated that betaine can upregulate *PPARG* (peroxisome proliferator-activated receptor γ) and its target genes (e.g., *aP2*, *FAS*, and *LPL*) by inhibiting the extracellular signal-regulated kinase 1/2 pathway, thereby facilitating fat accumulation [38]. In addition, betaine helps to increase the absorption of FAs and expression of several muscle fat transporters (such as FAT/CD36, FATP1, and FABP3) [39]. Actually, adding 2500 mg/kg betaine to the feed of DLY crossbred pigs can significantly increase IMF content [40]. In our study, the TP group had higher betaine levels and IMF content. Carnosine is a dipeptide composed of β-alanine and histidine, which is abundant in animal muscle tissue. Previous studies have suggested that carnosine affects the tenderness, juiciness, colour, and flavour of meat [41]. Carnosine enhances meat quality by regulating fat metabolism and antioxidant capacity, inhibiting the formation of methemoglobin, and reducing fat oxidation [42]. Here, we found that the TP group showed higher carnosine levels and superior meat quality, which is consistent with these metabolomic data. L-carnitine is a critical factor that affects the β-oxidation process of fatty acids. It also participates in the Transport of long-chain fatty acids. This transport process further promotes the oxidative degradation of fatty acids, ultimately promoting the formation of ATP [43]. A previous study suggests that L-carnitine may promote IMF deposition [44]. Adding L-carnitine to lamb feed can increase IMF content and improve meat quality. Another research showed that dietary supplementation with L-carnitine leads to elevated levels of MUFA and PUFA, while reducing the content of SFA [45]. We found that the TP group had higher carnitine levels and richer IMF content, which is consistent with these reports. The mechanism by which lysophosphatidylcholine regulates fat deposition in muscles is still unclear. During different growth stages of finishing pigs, intramuscular fat content in muscle showed a significant increasing trend with body weight gain. Meanwhile, the contents of C18:2 and C20:3n-3 fatty acid components in lysophosphatidylcholine within IMF also increased significantly [46]. This change is closely related to the shift in lipid metabolism in pigs from a “muscle growth-dominant” to a “fat deposition-dominant” state in the late finishing stage. This finding suggests lysophosphatidylcholine may be related to IMF deposition and deserves further study.

**Figure 7:**
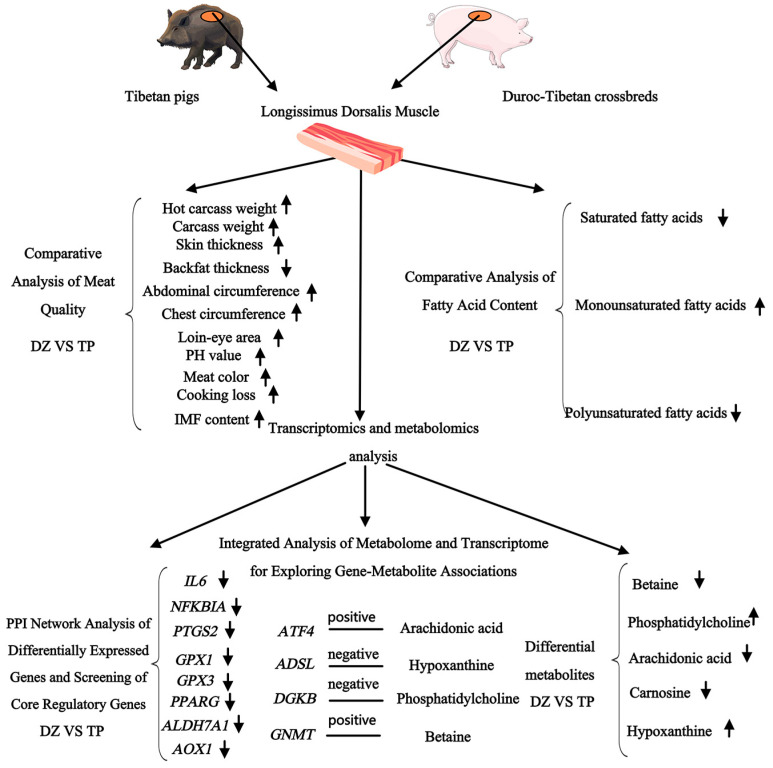
Schematic overview: From phenotypic traits to multi-omics analysis, comprehensively revealing the molecular mechanisms and pathways influencing meat quality. (Arrows pointing upward indicate a higher expression level, whereas arrows pointing downward indicate a lower expression level).

Combining transcriptome and metabolome analyses can provide a comprehensive understanding of the biological process from gene expression to metabolite production. KEGG analysis of transcriptomic and metabolomic data identified 20 enriched pathways. The DEGs and DMs were selected for Pearson correlation analysis. Correlation networks and clustering heatmaps were created to interpret the relationships between metabolites and genes. Activation of the GnRH receptor promotes lipid droplet accumulation in adipocytes by inhibiting the AMPK signalling pathway [47]. The *ATF4* gene and arachidonic acid are enriched in the GnRH pathway. ATF4 modulates lipid metabolism via lipolysis and lipogenesis, whereas arachidonic acid and its metabolites function as signalling molecules that impact the activity and functional role of *ATF4*, collectively aiding in the maintenance of lipid homeostasis [48]. Existing evidence suggests that these two components work together to influence lipid metabolism. It is inferred that *ATF4* and arachidonic acid jointly regulate fat deposition through the GnRH pathway. In this study, *ATF4* exhibited a positive correlation with arachidonic acid. Additionally, choline metabolism is closely associated with lipid metabolism. Choline influences lipid metabolism by regulating two classes of gene expression. These genes are involved in fatty acid synthesis (e.g., *ACC* and *FASN*) and those related to intracellular transport processes (e.g., LPL and CD36) [49]. Moreover, the enriched *DGKB* gene shows a significant negative correlation with the lysophosphatidylcholine within this pathway. Previous research has indicated that the *DGKB* gene encodes diacylglycerol kinase β (DGLKβ), which is involved in intracellular lipid metabolism [50]. *DGKB* phosphorylates diacylglycerol (DAG) into phosphatidic acid (PA), which participates in PC synthesis via the CDP–choline pathway. However, elevated *DGKB* expression reduces intracellular DAG storage, indirectly inhibiting the activation of the PKC signalling pathway [51]. The downregulated PKC activity inhibits phospholipase A_2_ activity, thereby decreasing lysophosphatidylcholine synthesis [52]. We inferred that the *DGKB* gene may regulate intramuscular fat deposition by modulating metabolites such as lysophosphatidylcholine 17:0, 20:4, 16:0 and 18:0, ultimately affecting IMF fat content. Within the glycine, serine, and threonine metabolic pathways, metabolites of these amino acids can influence fatty acid synthesis. For instance, glycine participates in methylation metabolism by generating methylene tetrahydrofolate (CH_2_-THF), which in turn affects fatty acid synthesis [49]. The metabolite of threonine, propionyl-CoA, is involved in the TCA cycle to provide precursors for fatty acid synthesis [53]. *GNMT*, a gene enriched within this pathway, displayed a significant positive correlation with the enriched metabolite betaine. Previous research has demonstrated that *GNMT* catalyzes the transfer of a methyl group from S-adenosylmethionine (SAM) to glycine, generating N-methylglycine (sarcosine) and S-adenosyl-L-homocysteine (SAH) as reaction products [49]. The sarcosine produced through this process can be further metabolized to form betaine. These reactions suggest that *GNMT* may regulate intramuscular fat deposition by modulating betaine levels. Furthermore, studies have shown that purine metabolism is closely linked to meat quality and sensory flavour. Within the purine metabolism pathway, the *ADSL* gene exhibited a negative correlation with the metabolite hypoxanthine. Additionally, another research finding has shown that *ADSL* is significantly and positively correlated with the inosine monophosphate (IMP) content [54]. IMP serves as the primary source of umami compounds in meat, participating in energy metabolism, pH regulation, and the storage of muscle glycogen. As an upstream substrate in purine metabolism, hypoxanthine enters the purine monophosphate pathway via hypoxanthine adenosine diphosphate succinate synthase (ADSS) and adenosine diphosphate succinate lyase (*ADSL*), ultimately forming adenosine monophosphate (AMP) [55]. High expression of *ADSL* will increase the activity of various enzymes and promote the conversion of AMP. As IMP is consumed, the cells will activate compensatory pathways to convert hypoxanthine into IMP. This allows hypoxanthine to be utilized more rapidly [55]. Therefore, it can be inferred that the *ADSL* gene influences meat flavour by regulating the levels of hypoxanthine. This study comprehensively reveals the molecular mechanisms and pathways influencing meat quality in genetic improvement, from phenotypic trait measurement to multi-omics analysis (Figure 7).

## 5. Conclusions and Prospects

In general, we conducted a comprehensive analysis of the meat quality, growth appearance, fatty acid content, IMF cell size, and transcriptome and metabolome data of DZs and TPs. We found differences in the texture of the meat between them. In terms of carcass morphology and slaughter traits, Duroc–Tibetan crossbred pigs exhibit hybrid vigour by combining favourable traits from both parental lines: the rapid growth performance of Duroc pigs and the superior meat quality characteristics of TP. Compared with foreign three-way crossbred pigs, Duroc–Tibetan crossbreeds display higher intramuscular fat content. Fatty acid profile analysis reveals that both pork types are predominantly composed of unsaturated fatty acids, conferring greater nutritional health benefits. Transcriptome analysis revealed that several genes exhibited high expression specifically in DZ, including *ATF4*, *ADSL*, *IL6*, and *DGKB*. These genes are related to lipid metabolism. Metabolomic analysis also revealed significant changes in specific fatty acids and metabolites in DZ, including betaine, carnosine, L-carnitine, and lysophosphatidylcholine. A joint analysis of transcriptome and metabolome data confirmed the relationship between these genes and metabolites, clarifying the molecular mechanism underlying the improvement of meat quality in Duroc–Tibetan crossbreeds. The elevated expression of the *ATF4* gene was strongly associated with arachidonic acid accumulation. The high expression of *DGKB* is associated with the reduction of LysoPC (17:0), (20:4), (16:0), and (18:0). The increased *GNMT* level was strongly associated with betaine accumulation. In contrast, *ADSL* gene expression showed a significant negative correlation with hypoxanthine. These associations may indicate adaptive modifications in lipid metabolism. By analyzing 8 differentially expressed genes related to lipid metabolism and differentially expressed metabolites, this study provides partial insights into the molecular basis underlying variety-specific fat deposition patterns. Future research will focus on in vitro and in vivo functional verification of these core genes to elucidate their specific function in regulating meat quality. Meanwhile, by combining epigenetics and gut microbiome analysis with the multi-dimensional regulatory network underlying the meat quality advantages of crossbred pigs, we aim to gain a deeper understanding of these mechanisms.

## Figures and Tables

**Table 1:** Determination of TP and DZ carcass and meat quality traits.

Items	TP	DZ
Hot carcass weight	38.93 ± 1.99	66.4 ± 13.02 *
Carcass weight, kg	25.51 ± 4.68	43.9 ± 8.17 *
Skin Thickness, mm	3.92 ± 0.31	4.05 ± 0.26
Backfat thickness, mm	21.93 ± 5.01	12.82 ± 5.55 *
Abdominal circumference, cm	85.99 ± 11.33	103.32 ± 7.49 *
Chest circumference, cm	75.11 ± 7.26	89.35 ± 4.69 *
Loin-eye Area, cm^2^	17.11 ± 3.08	40.65 ± 2.76 *
pH value after 45 min	6.18 ± 0.11	6.17 ± 0.03
pH value after 24 h	5.51 ± 0.01	5.86 ± 0.07 *
Lightness in meat color (L*)	49.42 ± 14.16	45.59 ± 8.65 *
Redness in meat color (a*)	14.54 ± 5.26	11.53 ± 5.42 *
Yellowness in meat color (b*)	6.91 ± 0.63	5.71 ± 2.03 *
Cooking Loss, %	36.83 ± 2.79	32.41 ± 2.01 *
IMF content, %	3.73 ± 0.13	2.20 ± 0.41 *

* indicates a significant difference (*p* < 0.05) between the TP and DZ groups.

## Data Availability

The raw datasets used and analyzed during the current study are available from the corresponding author upon reasonable request.

## Supplementary Materials

The following supporting information can be downloaded at: https://www.mdpi.com/article/10.3390/ani16020214/s1. Table S1. Primer information of qPCR. Table S2. Profile of the transcriptome sequence data. Table S3. KEGG Enrichment Analysis Pathways for Combined Transcriptome and Metabolome Data of Intramuscular Fat in TP and DZ Muscles. Table S4. Feed formula of pigs.
