# Supplementary material for: Multi-Omics Integration Reveals Key Genes, Metabolites and Pathways Underlying Meat Quality and Intramuscular Fat Deposition Differences Between Tibetan Pigs and Duroc × Tibetan Crossbred Pigs
Source: Animals (Basel). 2026 Jan 11;16(2):214. doi: 10.3390/ani16020214 (PMC12838071; doi:10.3390/ani16020214)
Supplement: Supplementary file 1 [file animals-16-00214-s001.zip › animals-4042325-supplementary.pdf]

Supplementary

Supplementary

Table S1. Primer information of qPCR

Gene	Primer Sequences (5′-3′)	Product Size
ALDH7A1	F:TCTCTATGGGCCACTCCACA<br>R:AGGGCGATCCATAACCTTGC	115 bp
AOX1	F:ACGAAAATGCTTCAGGTGGC<br>R:TTACAGCCAACCCGTTGAGG	142 bp
GPX1	F:CAACCAGTTTGGACATCAGGAAA<br>R:CGAAGAGCGGGTGAGCATTT	146 bp
GPX3	F:GGGCCAGTACGTTGAACTGA<br>R:GAGTTGTCTCCCGGTTCCTG	111 bp
IL6	F:TCTGGGTTCAATCAGGAGACC<br>R:ATCTGCACAGCCTCGACATT	125 bp
NFKBIA	F:ACCAACTACAATGGCCACAC<br>R:CAGCACCCAAAGACACCAAC	88 bp
PPARG	F:ATGCTGTCATGGGTGAAACTCT<br>R:ACCATGGTCACCTCTTGTGA	100 bp
PTGS2	F:AGCAGGCTGATACTGATAGGAGA<br>R:AGCAGCTCTGGGTCAAACTT	104 bp
ACTIN	F:CCAGAGCAATCAGGGACC<br>R:CAATGGACGGGAAAACAGCC	146 bp

Table S2. Profile of the transcriptome sequence data

Items	JNDZ1	JNDZ2	JNDZ3	JNTP1	JNTP2	JNTP3
Rawreads (million)	58.82	65.85	60.17	45.77	46.83	51.19
Clean reads (million)	58.18	65.32	59.71	45.35	46.41	50.46
Error rate(%)	0.0244	0.0245	0.0244	0.024	0.024	0.0254
Q20(%)	98.24	98.24	98.24	98.4	98.42	97.85
Q30(%)	94.75	94.73	94.75	95.21	95.25	93.89
GC content(%)	51.03	51.78	51.31	51.39	51.09	50.21

Table S3. KEGG Enrichment Analysis Pathways for Combined Transcriptome and Metabolome Data of Intramuscular Fat in TP and DZ Muscles

KEGG pathway	Gene Count	Gene Annotation	Meta Count	Meta Annoation
Diabetic cardiomyopathy	8	NDUFA6、NCF4、NCF1、MAPK10、AGT、PPIF、SLC25A4、GP91-PHOX	1	L-Carnitine
Purine metabolism	11	ADSL、PDE4B、ENTPD8、PDE7A、PAICS、NUDT2、ATIC、ADCY2、NUDT16、CANT1、AK8	1	Hypoxanthine
Thermogenesis	7	NDUFA6 、 CREB3L4 、 SLC25A0 、 PPARG 、 PNPLA2 、 RPS6KB2 、 ADCY2	1	L-Carnitine
Choline metabolism in cancer	5	SLC22A3 、 MAPK10 、 RPS6KB2 、 DGKB 、 EIF4EBP1	4	LysoPC(20:4(5Z,8Z,11Z,14Z)) 、 LysoPC(16:0) 、 LysoPC(17:0) 、 LysoPC(18:0)
Aldosterone synthesis and secretion	6	CACNA1I 、 ATF4 、 CREB3L4、AGT、ADCY2、TMEM52	1	Arachidonic acid
Arachidonic acid metabolism	7	CYP2B6B 、 AKR1C2 、 PTGS2、PLAAT3、GPX1、GPX3	1	Arachidonic acid
Pyrimidine metabolism	7	ENTPD8 、 CMPK2 、 NUDT2、TK1、CANT1、UCK2、TYMS	1	Cytidine
Regulation of lipolysis in adipocytes	5	ABHD5、PNPLA2、PTGS2、ADCY2、PLAAT3	1	Arachidonic acid
Glycine, serine and threonine metabolism	3	ALDH7A1、GNMT、SHMT1	1	Betaine

Table S4. Feed formula of pigs

Items	%
Corn	62.00
Soybean meal	25.80
Rice bran meal	8.20
Wheat middling	1.00
Soybean oil	1.00
Vitamin－mineral premix	2.00
